# Comparative transcriptomic analysis reveals gene regulation mediated by caspase activity in a chordate organism

**DOI:** 10.1186/s12860-021-00388-0

**Published:** 2021-10-06

**Authors:** Gabriel Krasovec, Anthi Karaiskou, Éric Quéinnec, Jean-Philippe Chambon

**Affiliations:** 1 Institut de Systématique, Evolution, Biodiversité (ISYEB), UMR 7205, Sorbonne Université, Muséum National d’histoire Naturelle, CNRS, EPHE, 7 Quai St-Bernard, F-75252 Paris Cedex 05, France; 2 Center for Chromosome Biology, School of Natural Sciences, National University of Ireland Galway, Galway, Ireland; 3 INSERM UMRS_938, Centre de recherche Saint-Antoine (CRSA), Sorbonne Université, Paris, France; 4 Centre de Recherche de Biologie Cellulaire de Montpellier (CRBM), Montpellier Univ., CNRS, 34000 Montpellier, France

**Keywords:** Comparative transcriptomics, Caspases, Apoptosis, Metamorphosis, Migration, Ascidians

## Abstract

**Background:**

Apoptosis is a caspase regulated cell death present in all metazoans defined by a conserved set of morphological features. A well-described function of apoptosis is the removal of excessive cells during development and homeostasis. Recent studies have shown an unexpected signalling property of apoptotic cells, affecting cell fate and/or behaviour of neighbouring cells. In contrast to the apoptotic function of cell elimination, this new role of apoptosis is not well understood but seems caspase-dependent. To deepen our understanding of apoptotic functions, it is necessary to work on a biological model with a predictable apoptosis pattern affecting cell fate and/or behaviour. The tunicate *Ciona intestinalis* has a bi-phasic life cycle with swimming larvae which undergo metamorphosis after settlement. Previously, we have shown that the tail regression step during metamorphosis, characterized by a predictable polarized apoptotic wave, ensures elimination of most tail cells and controls primordial germ cells survival and migration.

**Results:**

We performed differential transcriptomic analysis between control metamorphosing larvae and larvae treated with the pan-caspase inhibitor Z-VAD-fmk in order to explore the transcriptional control of apoptotic cells on neighbouring cells that survive and migrate. When caspase activity was impaired, genes known to be involved in metamorphosis were downregulated along with other implicated in cell migration and survival molecular pathways.

**Conclusion:**

We propose these results as a confirmation that apoptotic cells can control surrounding cells fate and as a reference database to explore novel apoptotic functions in animals, including those related to migration and differentiation.

**Supplementary Information:**

The online version contains supplementary material available at 10.1186/s12860-021-00388-0.

## Background

Apoptosis is a regulated cell death defined by morphological features and depending on caspases [1–3]. Being ubiquitous in metazoans [4–7], the main function of apoptosis is the elimination of unwanted cells [8], such as the interdigital tissues during embryogenesis of tetrapods [9] or larval tissues during metamorphosis of many animals [10]. Apoptosis is also implicated in homoeostasis by fine-tuning the balance between cell death and proliferation like in gonad size control in rodents [11].

While the function of cell elimination by apoptosis is highly documented, apoptosis seems to have a broader morphogenetic function that goes beyond cells elimination. Accumulating evidence in many metazoans suggest that apoptotic cells can emit caspase-dependent signals to their neighbours, modulating their fate (survival/death, differentiation or proliferation) or behaviour (migration) [12, 13]. Apoptotic-induced proliferation was reported in several organisms, such as in the cnidarian *Hydra*, where apoptosis promotes proliferation of adjacent cells through emission of Wnt3 signalling in a caspase-dependant manner during head regeneration [14]. Similar observations have been made during rodent liver regeneration, where cleavage of iPLA2 by caspases in apoptotic cells led to proliferation of adjacent cells [15]. Apoptotic cells can also induce differentiation, as was reported during the metamorphosis of the cnidarian *Hydractinia echinata* [16]*.* Finally, apoptotic cells modulate death or survival balance, as was observed in the *Drosophila* imaginal disc, by producing the Tie ligand Pvf1, that induces death resistance of neighbouring cells [17]. Apoptotic-dependent migration is well described in mammals, in which apoptotic cells secrete directional signals such as Lysophophatidylcholine or Sphingosine-1-Phosphate, promoting migration of leukocytes [18, 19]. Furthermore, in *Xenopus laevis* tadpole, an apoptotic-dependent axon guidance was suggested during tail regeneration after injury [20].

While caspase-dependent signals emitted by apoptotic cells start to be characterised, almost no target genes are identified in receptor cells. Evaluating the capacity of caspases to affect gene expression and influence fate and/or behaviour of cells adjacent to apoptosis is the first fundamental step to better characterise this new function in animals.

In this report, we took advantage of the predictable spatiotemporal apoptotic profile of ascidians [21, 22], in particular during the *Ciona intestinalis* metamorphosis, which is concomitant with cell survival and cell migration [21, 23, 24]. *Ciona* belongs to the Urochordata, the sister group of vertebrates [25], and has a biphasic life cycle composed of a pelagic larva and a benthic adult. Embryogenesis generates a tadpole-like swimming larva consisting of an anterior trunk and a posterior tail which, after few hours of swimming, settles and metamorphoses into a sessile juvenile. A hallmark of metamorphosis is the apoptotic-dependent tail regression [21] during which apoptosis starts at the tip of the tail and propagates toward the trunk, making it easily anticipated; a unique feature among chordates. Using TUNEL labelling and electron microscopy, we previously described in detail this apoptotic profile. Apoptosis starts in epidermal cells at the most posterior tail tip, then, it affects the notochord cells and the striated muscle cells in a postero-anterior wave. The tunic cells are also randomly affected. On the opposite, two tissues survive and migrate during, or prior to, tail regression; the ventral endodermal strand and the eight primordial germ cells (PGC) [23, 24, 26]. PGC, located at the postero-ventral side of the tail tip, escape from cell death by moving toward the trunk, and are consequently surrounded by apoptotic cells during the tail regression. Interestingly, we previously demonstrated that the pan-caspase inhibitor Z-VAD-Fmk blocks both apoptotic dependent-tail regression and PGC migration, suggesting that PGC migration is regulated by apoptotic cells [23].

To identify genes controlled by caspases activity during tail regression, we performed a comparative transcriptomic analysis at the beginning and at mid-tail regression between control metamorphosing larvae or larvae exposed to the pan-caspase inhibitor Z-VAD-fmk, already successfully used in *Ciona* [21] (Fig. S1). Based on a de novo transcriptome, we identified 61 genes differentially expressed between the beginning and mid-tail regression in control larvae. Next, we found 65 genes differentially expressed between control and treated larvae (21 and 44 at the beginning and at mid-tail regression, respectively). Interestingly, misregulation of some genes upon caspase inhibition was reported during ascidian metamorphosis, but also reported in cell survival and cell migration in several animals [27–30]. Our results offer a first database of genes transcriptionally modulated by apoptosis during a dynamic morphogenetic process in a chordate, allowing exploration of novel apoptotic functions in animals.

## Results

### Genes differentially express between the onset and the mid-tail regression

**Fig. 1:**
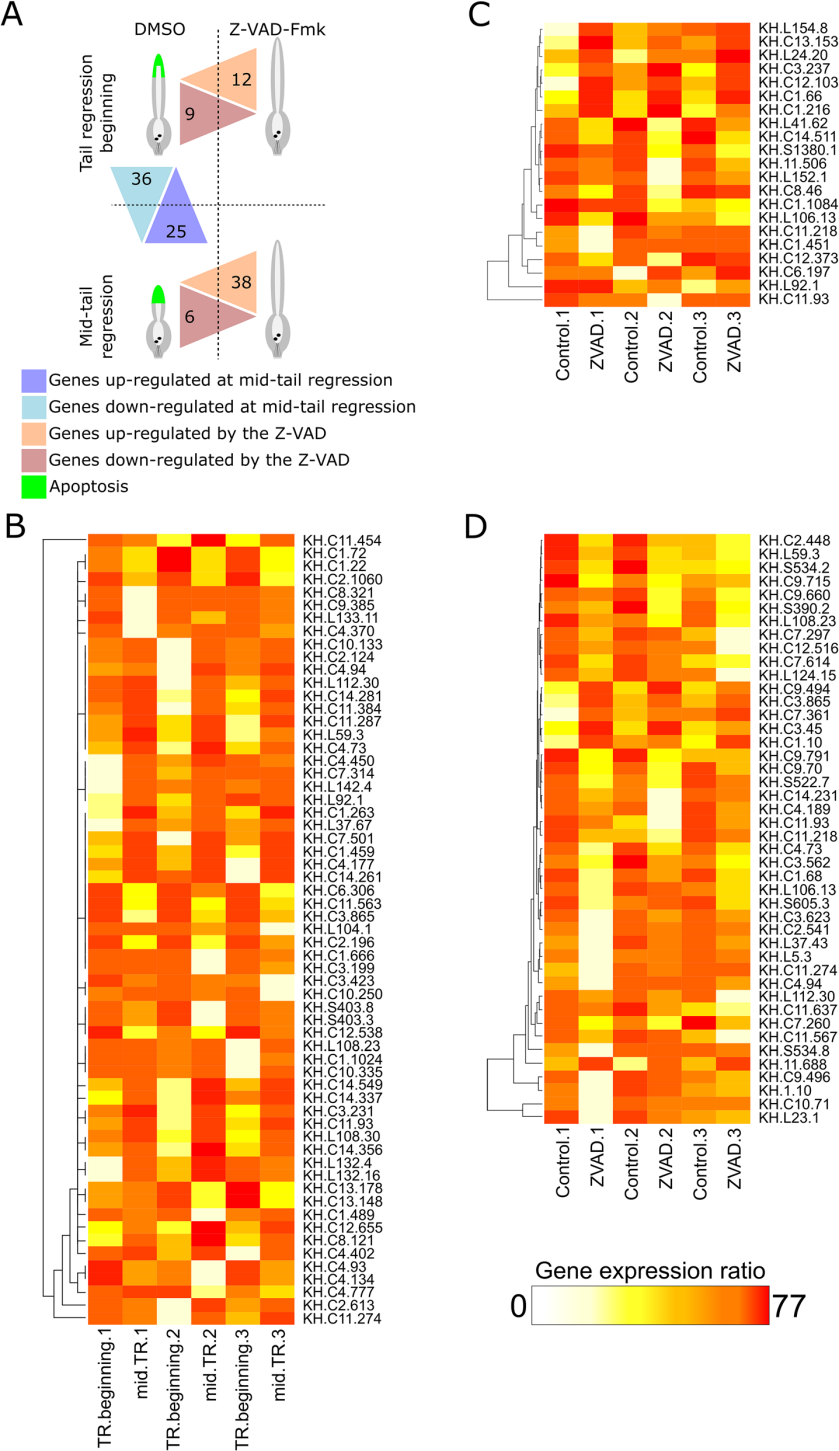
**A**, Counting of genes differentially expressed between conditions. **B**, Heatmaps showing genes differentially expressed in control between the beginning of tail regression (TR) and the mid-regression. **C**, Heatmaps showing genes diffenrentially expressed at the beginning of tail regression between control and Z-VAD-fmk treated larvae. **D**, Heatmaps showing genes diffenrentially expressed at mid-regression between control and Z-VAD-fmk treated larvae

**Fig. 2:**
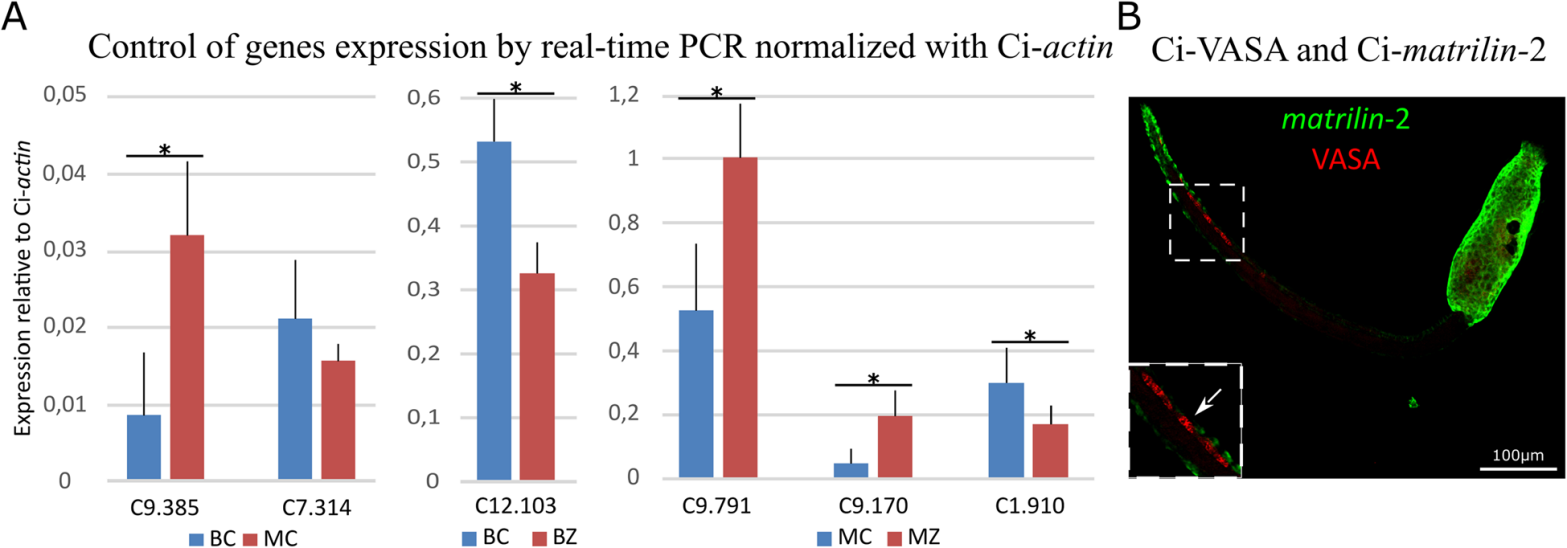
**A**, Control of gene expression by real-time PCR. Real-time PCR results are consistent with our differential transcriptomic analysis, with however expression level of KH.C7.314 between beginning and mid-tail regression of control larvae not statistically significant. BC: Tail regression beginning, control. BZ: Tail regression beginning, Z-VAD. MC: Mid-tail regression, control. MZ: Mid-tail regression, Z-VAD. * indicates statistically significant differences. **B**, *In situ* hybridization of KH.L132.4 (*matrilin-2*) gene (in green) combined with immunnodection of Ci-VASA to detect PGC (in red) in premetamorphic larva. *Matrilin-2* is expressed in epidermal cells at the tip of the tail, the most anterior expressing epidermal cells are in contact with PGC (higher magnification, white square). *Matrilin-2* expression is also detected in the trunk epidermis. White square higher magnification of the PGC region

Globally, the robustness of our analysis is shown by a heat map allowed visualization of result congruence between replicates (Fig. 1). To confirm relevance of our data, we randomly chose 6 genes differentially expressed in our transcriptomic analysis to performed real-time PCR (Fig. 2A). Five of them exhibited an expression profile that was similar for both experiments.

One of them, KH.C7.314, is less expressed at the mid-tail regression in comparison to the beginning of the process in control larvae in our transcriptomic analysis. Similar profile was observed with real-time PCR, but the difference is less pronounced and not statistically significant. Despite this, control by real-time PCR globally validated our transcriptomic data.

First, we have focused on genes differentially expressed in control metamorphosing larvae between the beginning and the mid-tail regression. Twenty-five genes (41%) were up-regulated from the beginning to the mid-tail regression with the strongest increase recorded for KH.C9.385 (*smyd3*) (Table 1). Conversely, 36 genes (59%) show decreased expression, with a subset of genes whose expression was no longer detectable such as KH.14.261 (*lysozyme*-like) (Table 2). Among these identified genes, several were already reported during a previous cDNA chip analysis of *Ciona* metamorphosis, such as KH.C7.314 (*MED30*), KH.C11.274 (*p-selectin*, known as *Ci-sushi*) which is expressed in almost all the tail, or KH.L132.4 (*matrilin-2*) expressed in epidermal cells at the tip of the tail in premetamorphic larva (Fig. 2B) [29]. In addition, some of the genes identified by our screen had already been detected by substantial RNA sequencing data coupled with *in situ* hybridization data available on ANISEED database (http://www.aniseed.cnrs.fr). For example, KH.L132.16 (*fibrillin-3*) starts to be expressed at mid-neurulae with a maximum expression in larva, in which it is detected in all the epidermis including the tail. Furhermore, KH.C10.250 (*fox-1*, RNA-binding) is expressed in tail muscle at mid-tailbud stage to become ubiquitous in larva [31].

**Table 1:** Genes upregulated at mid-tail regression in comparison to the beginning

KyotoGrail KH gene model	Expression ratio	Identity and/or GO function	References in ascidians/reported functions in animals
KH.C9.385	77,151	histone-lysine N-methyltransferase SMYD3	x
KH.C3.865	64,573	orphan gene	x
KH.C6.306	33,673	glutamate receptor ionotropic, kainate 2 (GRIK2)	x
KH.C11.563	31,576	Breast cancer metastasis-suppressor 1	x
KH.L104.1	19,902	unnamed protein product	x
KH.C3.199	17,833	26S proteasome regulatory subunit (RPT4B)	x
KH.C1.666	13,954	uncharacterized LOC100178010	x
KH.C8.321	12,169	S-adenosyl-L-methionine-dependent methyltransferase	x
KH.C10.250	7367	Fox-1, RNA-binding	x
KH.C2.196	5222	Integrin alpha N-terminal domain	migration
KH.C3.423	4798	At3g29763	x
KH.S403.3	4672	Periplasmic binding protein-like II, UBX domain-containing protein 1	x
KH.S403.8	4672	RSL1D1	x
KH.C12.538	2968	V-type proton ATPase subunit F-like (ATP6V1F)	x
KH.L133.11	2668	protein 248-like; ubiquitine like	x
KH.C4.370	2541	ADAMTS-like protein	x
KH.C1.489	2389	serpin H1; serpin A5	x
KH.C4.134	2290	Spermadhesin; MAM and LDL-receptor class A	x
KH.C4.93	2270	Zonadhesin; MAM and LDL-receptor class A	x
KH.C2.1060	2210	uncharacterized protein LOC100177726	x
KH.C1.22	2196	ubiquitin-conjugating enzyme E2 D3-like (UBE2D3)	x
KH.C4.777	2134	sushi, EGF and pentraxin domain-containing protein 1-like	x
KH.C1.72	2096	zinc finger protein Ap-Zic	x
KH.C13.148	2016	cochlin	x
KH.C13.178	2006	uncharacterized protein LOC100185406	x

**Table 2:** Genes downregulated at mid-tail regression in comparison to the beginning

KyotoGrail KH gene model	Expression ratio	Identity and/or GO function	References in ascidians/reported functions in animals
KH.C14.261	0,023	putative lysozyme-like protein; PE-PGRS family protein	x
KH.L37.67	0,025	TGM1, Transglutaminase	x
KH.C7.501	0,026	ferritin	x
KH.C1.459	0,026	Sorting nexin-18	x
KH.C4.177	0,028	Glucocorticoid receptor-like, Pinch protein	x
KH.C7.314	0,050	mediator of RNA polymerase 2 transcription subunit 30 (med30)	Chambon et al. 2007 (ci0100134193) apoptosis
KH.C14.281	0,050	glycerol-3-phosphate (1)-acyltransferase	x
KH.L142.4	0,056	universal stress protein YxiE	x
KH.C1.263	0,061	ribosomal protein L4	x
KH.C10.335	0,061	calmodulin like protein	migration
KH.C11.384	0,092	uncharacterized protein LOC100186758	x
KH.L112.30	0,103	transmembrane matrix receptor MUP-4-like	x
KH.C1.1024	0,129	glutamate-rich WD repeat-containing protein 1	x
KH.C4.94	0,129	lipopolysaccharide-induced tumor necrosis factor-alpha factor	x
KH.L92.1	0,144	protein DDB_G0283697	x
KH.C2.124	0,155	Glycerol-3-phosphate (1)-acyltransferase	x
KH.L108.23	0,167	protein DD3–3	x
KH.C11.93	0,174	N-acetyltransferase 6	x
KH.C10.133	0,194	holine-phosphate cytidylyltransferase A-like	x
KH.C3.231	0,216	uncharacterized protein LOC100184171	x
KH.L59.3	0,227	uncharacterized protein LOC100175851	x
KH.C4.450	0,235	sushi, von Willebrand factor type A, EGF and pentraxin domain-containing protein 1	x
KH.C11.274	0,243	P-selectin-like	Chambon et al. 2007 (ci0100139289) apoptosis
KH.C4.73	0,268	uncharacterized protein LOC100175287	x
KH.C14.356	0,275	putative lysozyme-like protein	x
KH.C11.287	0,278	unc-93 homolog A-like	x
KH.C14.337	0,313	lysozyme-like protein; PE-PGRS family protein PE_PGRS30	x
KH.C14.549	0,335	lysozyme-like protein; PE-PGRS family protein PE_PGRS30	x
KH.L108.30	0,344	uromodulin-like 1 precursor	migration
KH.C12.655	0,390	uncharacterized protein LOC113474772	x
KH.L132.16	0,428	fibrillin-3; MATN2	migration
KH.L132.4	0,428	matrilin-2; MEGF6	Chambon et al. 2007 (ci0100138016) migation
KH.C2.613	0,443	zinc transporter 9-like	x
KH.C11.454	0,446	uncharacterized protein LOC100184022	x
KH.C8.121	0,463	SCO-spondin	x
KH.C4.402	0,470	galanin receptor type 3	x

We also observed that some among these were also impacted by Z-VAD-fmk treatment (Tables 3, 4, 5 and 6) such as KH.C6.197 (*meta2*), KH.C1.10 (*LIM*), KH.C1.216 (*myosin light chain *3), and KH.C11.274 (*Ci-sushi*) [27–30]. The detection of genes already known to be expressed in the tail and involved in tail regression (i.e. *Ci-sushi*, *Ci-meta2*) is consistent with previous studies on ascidians metamorphosis, validates our experimental procedures and supports the relevance of our database.

**Table 3:** Genes upregulated by the Z-VAD-fmk at the tail regression beginning

KyotoGrail KH gene model	Expression ratio	Identity and/or GO function	References in ascidians/reported functions in animals
KH.C11.506	9850	girdin	apoptosis
KH.S1380.1	7405	mucin-5 AC	apoptosis
KH.C8.46	4453	vesicle-associated membrane protein 4-like	x
KH.C1.451	4028	orphan gene	x
KH.C14.511	3917	myotubularin-related protein 2-like	x
KH.L41.62	3503	reticulocalbin-2-like	x
KH.L152.1	3305	proline-rich protein PRCC	x
KH.C11.93	2521	gamma-glutamylcyclotransferase 2; N-acetyltransferase 6	x
KH.C11.218	2463	cytospin-A isoform X1	x
KH.C1.1084	2290	thioredoxin-2-like	apoptosis
KH.L106.13	2121	DENN domain-containing protein 5B-like	x
KH.C12.373	2011	uncharacterized protein LOC100175595	x

**Table 4:** Genes downregulated by the Z-VAD-fmk at the tail regression beginning

KyotoGrail KH gene model	Expression ratio	Identity and/or GO function	References in ascidians/reported functions in animals
KH.L92.1	0,473	protein DDB_G0283697	x
KH.C6.197	0,449	META2 protein precursor	Nakayama et al., 2001–2002; Chambon et al., 2007
KH.C1.216	0,369	myosin light chain 3, skeletal muscle	Nakayama et al., 2002
KH.L24.20	0,366	collagen alpha-6(VI) chain-like	migration
KH.L154.8	0,309	unnamed protein product	x
KH.C13.153	0,308	uromodulin-like	migration
KH.C3.237	0,229	60S ribosomal protein L7a	x
KH.C12.103	0,098	nuclear lamin-L (II)-like	x
KH.C1.66	0,000	orphan gene	x

**Table 5:** Genes upregulated by the Z-VAD-fmk at mid-tail regression

KyotoGrail KH gene model	Expression ratio	Identity and/or GO function	References in ascidians/reported functions in animals
KH.C12.516	11,357	neutral ceramidase-like	Kassmer et al. (2015) migration
KH.C9.170	10,571	protein CNPPD1	x
KH.C2.541	7634	cell cycle control protein 50A	x
KH.C3.623	7551	kinesin-like protein 2	x
KH.C4.189	6713	ribosomal protein CEP52	x
KH.L124.15	5285	vesicular integral-membrane protein VIP36	x
KH.S522.7	5242	TPA: zinc finger protein	x
KH.C14.231	4625	60S ribosomal protein L12	x
KH.S605.3	4398	gamma-crystallin S	x
KH.C9.791	4198	integrin alpha-2-like	migration
KH.C4.73	4127	uncharacterized protein LOC100175287	x
KH.L5.23	4122	uncharacterized protein LOC100179749	x
KH.C1.568	3255	uncharacterized protein LOC100176279	x
KH.S390.2	3227	RNA-binding motif, single-stranded-interacting protein 1	x
KH.C9.660	3187	supervillin-like	migration
KH.L106.13	3179	DENN domain-containing protein 5B-like	x
KH.S534.8	3075	coiled-coil domain-containing protein 178-like	x
KH.L108.23	2846	protein DD3–3	x
KH.C7.614	2824	SLIT and NTRK-like protein 3	x
KH.C2.448	2822	plasminogen	x
KH.C7.297	2732	solute carrier family 22 member 21	x
KH.L37.43	2586	L-threonine ammonia-lyase	x
KH.C11.274	2571	P-selectin-like	Chambon et al., 2007 (ci0100139289) apoptosis
KH.C11.218	2518	cytospin-A	x
KH.L59.3	2488	uncharacterized protein LOC100175851	x
KH.C10.71	2418	3-phosphoinositide-dependent protein kinase 1	x
KH.C9.715	2397	neurobeachin-like protein 1	x
KH.C3.562	2392	unconventional myosin-X	x
KH.C4.94	2374	lipopolysaccharide-induced tumor necrosis factor-alpha factor	x
KH.S534.2	2333	RB1-inducible coiled-coil protein 1	x
KH.C9.496	2327	uncharacterized protein LOC100183253	x
KH.C1.10	2314	LIM and SH3 protein	Terasaki et al., 2008
KH.C11.93	2252	N-acetyltransferase 6	x
KH.L112.30	2179	transmembrane matrix receptor MUP-4-like	x
KH.C11.567	2162	serine palmitoyltransferase 2	x
KH.L23.11	2094	uncharacterized protein LOC100178692	x
KH.C11.637	2056	putative glutathione-specific gamma-glutamylcyclotransferase 2	x
KH.C7.260	2031	tropomyosin-like protein	Nakayama et al., 2002 migration

**Table 6:** Genes down regulated by the Z-VAD-fmk at mid-tail regression

KyotoGrail KH gene model	Expression ratio	Identity and/or GO function	References in ascidians/reported functions in animals
KH.C11.688	0,385	thioredoxin-related transmembrane protein 1-like	x
KH.C9.494	0,319	myosin light chain kinase, smooth muscle	Nakayama et al., 2002 migration
KH.C3.865	0,270	orphan gene	x
KH.C7.361	0,237	regulator of chromosome condensation 1 (rcc1)	x
KH.C3.45	0,217	protein kinase C alpha type	Apoptosis
KH.C1.910	0,109	low-density lipoprotein receptor-related protein 2	x

### Caspase inhibition affects gene expression during the tail regression

In total, 65 genes were affected by the inhibition of caspases during *Ciona* tail regression (Fig. 1). At the beginning of tail regression 12 genes were upregulated by the Z-VAD-fmk treatment (so negatively regulated by caspase activity in physiological conditions) with the greatest expression increase observed for KH.C11.506 (*girdin*) and KH.S1380.1 (*mucin-5 AC*) (Table 3), and 9 genes were downregulated (so positively regulated by caspase activity) with extreme effects on KH.C1.66, which is no longer expressed (Table 4).

At mid-tail regression, we identified 38 upregulated genes in the Z-VAD-fmk condition with the highest increase noted for KH.C12.516 (Table 5), and 6 downregulated genes (Table 6) with strongest effect concerning KH.C1.910.

### Transcription is necessary for the PGCs migration

**Fig. 3:**
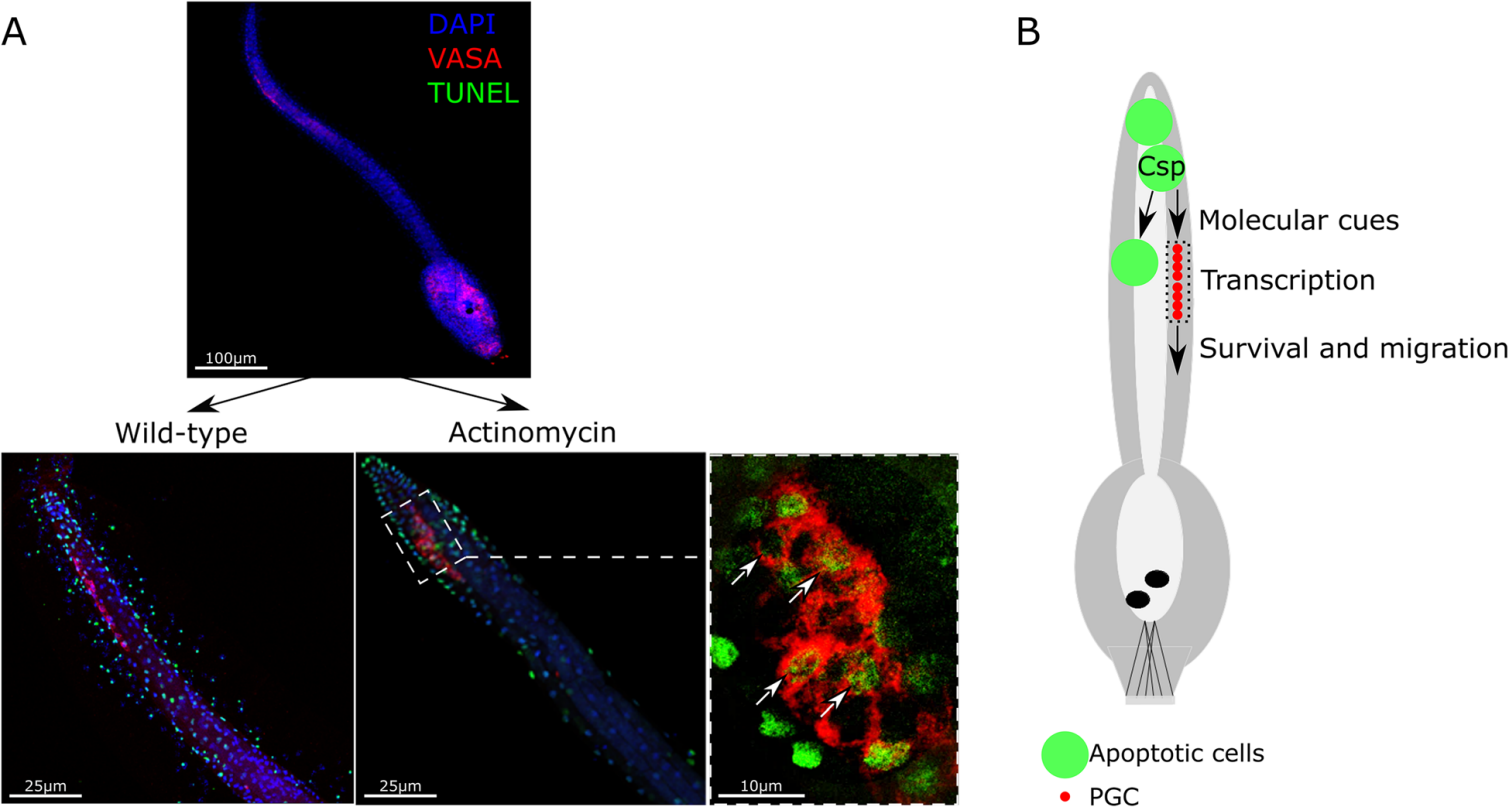
**A**, Larvae labelled by TUNEL (green) with VASA immunostaining (red) and counterstained with DAPI (blue). PGC of larvae treated with the transcription inhibitor actinomycin-D (1 μg/ml) show impaired migration and undergo apoptosis. **B**, Representation of apoptotic functions during tail regression. Apoptotic cells emit signals modifying transcription of adjacent cells leading to PGC survival and migration to the trunk

We previously demonstrated that Z-VAD-fmk blocked both tail regression and PGC migration, and that apoptotic wave propagation and PGC migration speed were correlated [23]. Here, we show that transcription is necessary for PGC survival and migration (Fig. 3). We exposed metamorphosing larvae to the transcription inhibitor actinomycin-D; in the control (devoid of actinomycin-D), a classical apoptotic profile is observed with PGC movement, as already described [23], and in actinomycin-D-treated larvae PGC migration is blocked and PGC nuclei become TUNEL-positive, indicating destruction as the apoptotic wave progresses (Fig. 3). This result, and our previous studies, argue that survival and migration of PGC depend on transcription that could be partially controlled by caspase activity from apoptotic cells (Fig. 3). The comparative transcriptomic analysis between control and Z-VAD-fmk treated larvae is fundamental to understand this apoptotic dependent migration.

## Discussion

### Caspase activity modulates expression of genes implicated in cell death/survival

Interestingly, the 2 genes most inhibited by caspase activity at the beginning of tail regression, *girdin* and *mucin-5 AC*, were both reported to promote survival in mammalian cells [32, 33]. Among the other genes negatively impacted by caspase activity, KH.C1.1084, the thioredoxin homologue in mammals, is able to control the activation of the executioner caspase 3, the initiator caspase 9, and the release of the cytochrome c, all characterising the intrinsic apoptosis [34–37]. In addition, KH.C3.45 (*kinase C alpha*) homologue is known to promote survival and increases anti-apoptotic Bcl-2 efficiency in cultured cells [38]. Taken together, our database highlights that cells expressed a variety of genes regulating cell death/survival fate in a caspase dependent manner, suggesting that apoptotic cells actively participate in apoptosis propagation during tail regression.

### Caspase activity modulates expression of genes implicated in cell migration

We identified numerous genes modulated by caspase activity, with a majority of them upregulated, previously observed in cell migration regulation. It is the case of KH.C1.216 (*myosin light chain 3*) and KH.C9.494 (*myosin light chain kinase*) for which homologues are key players in mesenchymal migration [32, 39] and KH.C13.153 (*uromodulin*-like) which provides guidance cues for neutrophile migration [40]. Furthermore, integrin subunits and components of the Extra Cellular Matrix, crucial for mesenchymal migration, are well represented in our transcriptomic approach. In addition, KH.C1.10 (*lasp1*) mammalian homolog is necessary for migration of NIH 3 T3 cells [41, 42].

Furthermore, KH.C12.516 (*ceramidase*) could be involved in tissue migration regulation, as it has been described as participating in the sphingolipid pathways. Interestingly, sphingosine 1-phosphate signalling leads to tissue migration [43], particularly in germline cells (due to to a sphingosine 1-phosphate receptor) in the tunicate *Botryllus* [44], making this pathway a strong candidate for PGC migration in *Ciona*. Given that these genes and PGC migration are both controlled by caspases [23], PGC migration can be regulated by apoptotic cells.

### Ascidians as crucial biological models to studies novel apoptotic function

This study, coupled with our previous work suggested that apoptosis regulates the migration of primordial germ cells [23], confirm *Ciona* as a perfect biological model to understand apoptotic-induced migration. In addition, a regulation of cell proliferation by apoptosis was reported in this species [45]. Ascidians are well known to have an impressive regenerative capacity for a chordate, in particular in the context of the reconstruction of the distal body parts after amputation, thanks to stem cell requisition from the branchial sac [46–48]. Apoptosis was reported to occur as a transient event at the site of injury, and precedes the regeneration of the neural complex, the oral siphon, or the atrial siphon [49]. In the case of siphon amputation, it has been shown by caspases inhibition experiment that an apoptotic-dependant Wnt signalling allows the cell proliferation, leading to the successful regeneration [45]. In addition, apoptosis appears crucial to maintain the growth of branchial sac and its inhibition lead to a hazardous organization of this tissue, suggesting a fundamental function in homeostasis maintaining. Importantly, stem cells requisition and migration from the branchial sac toward the wounding site seem also dependant on caspases, making it an other potential apoptotic-dependant migration [45]. Taken together, all of these studies make *Ciona* one of the rare biological models where several cell fates and behaviours, controlled by apoptotic cells in a caspases dependent manner, has been put in evidence or suggested in adults as well as in larvae.

In the colonial ascidian *Botryllus schlosseri*, apoptosis is known to play on budlets elimination [50–52]. In this species, blastogenetic cycle (asexual reproduction of adults) give rise to a bud, itself given next four budlets (reviewed in [53, 54]). Usually, two of them growth despite the two other undergo apoptosis and are reabsorbed. In addition to this function in the blastogenetic cycle, apoptosis was also reported in tunic, epidermis, branchial leaflet, vessel epithelium, circulatory system or gonads in adults. With so many tissues presenting apoptosis in physiological condition, it seems clear that apoptosis should have crucial functions in *Botryllus* and likely could participate to regulation of other cell fates and behaviours as it was reported in *Ciona*. All of these tissues are as many opportunities to deepen or understanding of apoptosis functions in a same species.

## Conclusions

Here, we offer a potential roadmap in order to design future functional studies on *Ciona* and other animals, including mammals, where apoptosis takes place together with other cell fates and behaviours. Identification of molecular actors needing cleavage for their activation, cell receptors receiving signals from apoptotic cells, or transcription factors could be promising for further research.

The last decade has brought evidence that the cleavage capacity of caspases is a prerequisite to activate molecular actors promoting migration or differentiation in a non-apoptotic context [55–59]. Consequently, Z-VAD-fmk blocks all processes led by caspases, making it crucial to distinguish the level of implication of apoptotic and non-dying cells for future research.

Finally, our study provides an additional and novel chapter in the research of apoptotic functions beyond their role as a simple cell elimination mechanism, opening opportunities in the field of medical science [60].

## Methods

### Sample collection

Adult *Ciona intestinalis* were collected in the field in Brittany (France) by the Centre National Marine Biological Resources, EMBRC-France, Roscoff (Station Biologique de Roscoff, Sorbonne Université, Brittany, France). Individuals were maintained in 35‰ salinity artificial seawater (ASW) at 18 °C in the UMR7138 Evolution Paris Seine laboratory (Sorbonne University, Paris, France). Oocytes and sperm were obtained by dissection and cross-fertilizations were performed in plastic Petri dishes. Embryos were cultured at 18 °C in 0.2 μm filtered ASW with 0.1 M Hepes (#H4034, Sigma-Aldrich; Merck KGaA). Embryos of each fertilisation were divided into two subsets before the first cell division to obtain one control and one treatment condition. Six fertilisations were performed, divided into twelve petri dishes (6 control with their respective 6 treatment conditions). After the treatment, larvae were collected at two time points (see below), and compacted by manual centrifugation before RNA extraction.

### Pharmacological treatments and TUNEL labelling

The pan-caspase inhibitor Z-VAD-Fmk (#V116, Sigma-Aldrich; Merck KGaA) was stored in DMSO (#D8418, Sigma-Aldrich; Merck KGaA) at − 20 °C and used at final concentration of 10 μM. After the settlement of more than 50% of larvae on the dish, and when some of them stopped moving (indicating that metamorphosis will begin soon), supernatant was discarded and replaced with filtered ASW containing DMSO as control, or Z-VAD-Fmk diluted in DSMO as treatment. Z-VAD-Fmk was renewed every two hours. The experiment was divided into two time points (with three biological replicates for each one – itself divided between control and treated larva) following control tail regression stages: first when the tail regression just began in the control (beginning), and the second at mid-progression (mid-tail regression) given three controls with three Z-VAD-Fmk equivalents for each point.

In a separate experiment, 1 μg/ml of actinomycin-D (#A1410, Sigma-Aldrich; Merck KGaA) was added to swimming larvae. After settlement and tail regression initiation, paraformaldehyde fixation, TUNEL labelling and Vasa immunostaining were performed as previously described [23].

### RNA preparation and sequencing

RNA was extracted using the RNAqueous™-Micro Total RNA Isolation Kit (#AM1931 – ThermoFisher Scientific) according to the manufacturer’s protocol. DNAse treatment was performed using the TURBO DNA-*free*™ Kit (#AM1907 - ThermoFisher Scientific). RNA was purified and concentrated using the RNeasy MinElute Cleanup Kit (#74204 – Qiagen) according to the manufacturer’s protocol. RNA quantity was measured using a NanoDrop and quality was assessed with the Experion RNA HighSens (#700–7155 – BioRad) from 1 μL (0.1 μL of RNA completed with 0.9 μL of Milli-Q water) following standard instructions. Transcriptomes were sequenced on Illumina Hi-Seq 2000 (150 bp reads) at the Sistemas Genomicos plate-form of the Ascires Biomedical Group (Spain).

### De novo transcriptome assembly and differential expression

Raw reads of the twelve *Ciona intestinalis* samples were checked with FastQC and merged to assemble a de novo transcriptome with Oases [61] and Trinity [62]. Best assemblies from each one (best N50) were merged with Cap3 to obtain the de novo transcriptome. De novo transcriptome contains 85,506 contigs (N50 = 1314, minimum contig length = 102, maximum contig length = 13,998). Then, raw reads of each sample were mapped against the de novo transcriptome with Bowtie2 [63] with standard parameters. Low quality mapping reads (MQ > 20) were removed using Samtools [64] and Picard Tools (http://broadinstitute.github.io/picard). Expression quantification was performed using HTSeq [65] and differential expression evaluated with the DESeq2 package from R. We kept the genes with an expression ratio - (control 1 + control 2 + control 3) / (treatment 1 + treatment 2 + treatment 3) – above 2 (upregulated) and lower than 0.5 (downregulated). Then, Gene Ontology (GO) enrichment was performed with Blast2GO 5.2 and sequences were blasted on the *Ciona* genome to identify their KyotoGrail KH gene model.

### Real-time PCR

We synthetised the cDNA from RNA coming from same sample which was sending for sequencing using the SuperScript™ II Reverse Transcriptase (18,064,014 - ThermoFisher Scientific) according to the manufacturer protocol. Real-time PCR was performed with the SYBR Green Supermix (Biorad) on a Biorad’s thermal cycler with the following profile: 95 °C for 10 min; 40 cycles of amplification with successively 95 °C for 15 s, 60 °C for 10s, and 72 °C for 20s; one cycle for melting curve analysis with an acquisition every 0.5 °C from 65 °C to 95 °C to verify the presence of a single product. Each assay included four successive dilutions as standard to determine the reaction efficiencies and Ct values. Reactions were done in triplicate. All PCR amplicon have a length from 140 to 160 bp. Normalized was made using the reference gene *Ci-actin* (forward 5′ ATGTGCAAGGCCGGTTT 3′; reverse 5′ GACACGGAGTTCGTTGT 3′), already successfully used [66]. Genes of interest and their primer pair are: KH.C9.385 with forward 5′ ACTTCTGAAAAGAGCGGACG 3′ and reverse 5′ CATTGCAACAGACCATCTTGC 3′, KH.C7.314 with forward 5′ AGCCAACTACCGAATGGGA 3′ and reverse 5′ GTTCCACGTCTCCAACTCTC 3′, KH.C12.103 with forward 5′ TGTCTGAGATACGAAAGCGT 3′ and reverse 5′ CATTTGCTCAAGATCGGCG 3′, KH.C9.791 with forward 5′ GGATCTAAGCACTGTTCTGGAC 3′ and reverse 5′ CAAGGGCGTTGGTGTTCAGT 3′, KH.C9.170 with forward 5′ AATCCCCGCCCTTGAAGAAA 3′ and reverse 5′ GGGGTGGATATGAATAACATGG 3′, and KH.C1.910 with forward 5′ ACATGAAGATTTTGGTACCGAC 3′ and reverse 5′ CAAAACTTGGCCAAAAAGTTGG 3′. Differences was evaluated by Wilcoxon Mann-Whitney tests using R i386 4.0.3 software, and considered statistically significant for *p*-value < 0.05.

### In situ hybridization combined with vasa immunnodetection

Larvae were fixed 2 h at 4 °C in MEM-PFA (4% paraformaldehyde, 0.1 M MOPS pH 7.4, 0.1 M NaCl, 1 mM EGTA, and 2 mM MgSO4, 0.05% Tween-20). After fixation larvae were washed three times and dehydrated through a graded series of ethanol/PBS baths and stocked in 100% ethanol at − 20 °C. Rehydratation was made by successive washes of ethanol/PBS solution to a full PBS final solution. Hybridization was made according to a previous protocol [67] except that methanol was replaced by ethanol. After hybridization, immunnodetection of VASA was conducted as we previously described [23].

## Supplementary Information

**Additional file 1: Supplementary Fig. 1.** Experimental design. Six independent fertilisations were performed, and each of them was divided into two batches, in twelve petri dishes. After settlement, supernatant was discarded and replaced with filtered ASW containing DMSO (control) or Z-VAD-Fmk diluted in DSMO (treatment). RNA sampling was done at two time points according to the control: immediately after observation of tail regression beginning, and at mid-tail regression.

## Data Availability

Any request can be addressed to the corresponding author. Raw data are available under BioProject PRJNA725676.
