# Supplementary figures and images for: Comparative transcriptomic analysis reveals gene regulation mediated by caspase activity in a chordate organism
Source: BMC Mol Cell Biol. 2021 Oct 6;22:51. doi: 10.1186/s12860-021-00388-0 (PMC8495957; doi:10.1186/s12860-021-00388-0)

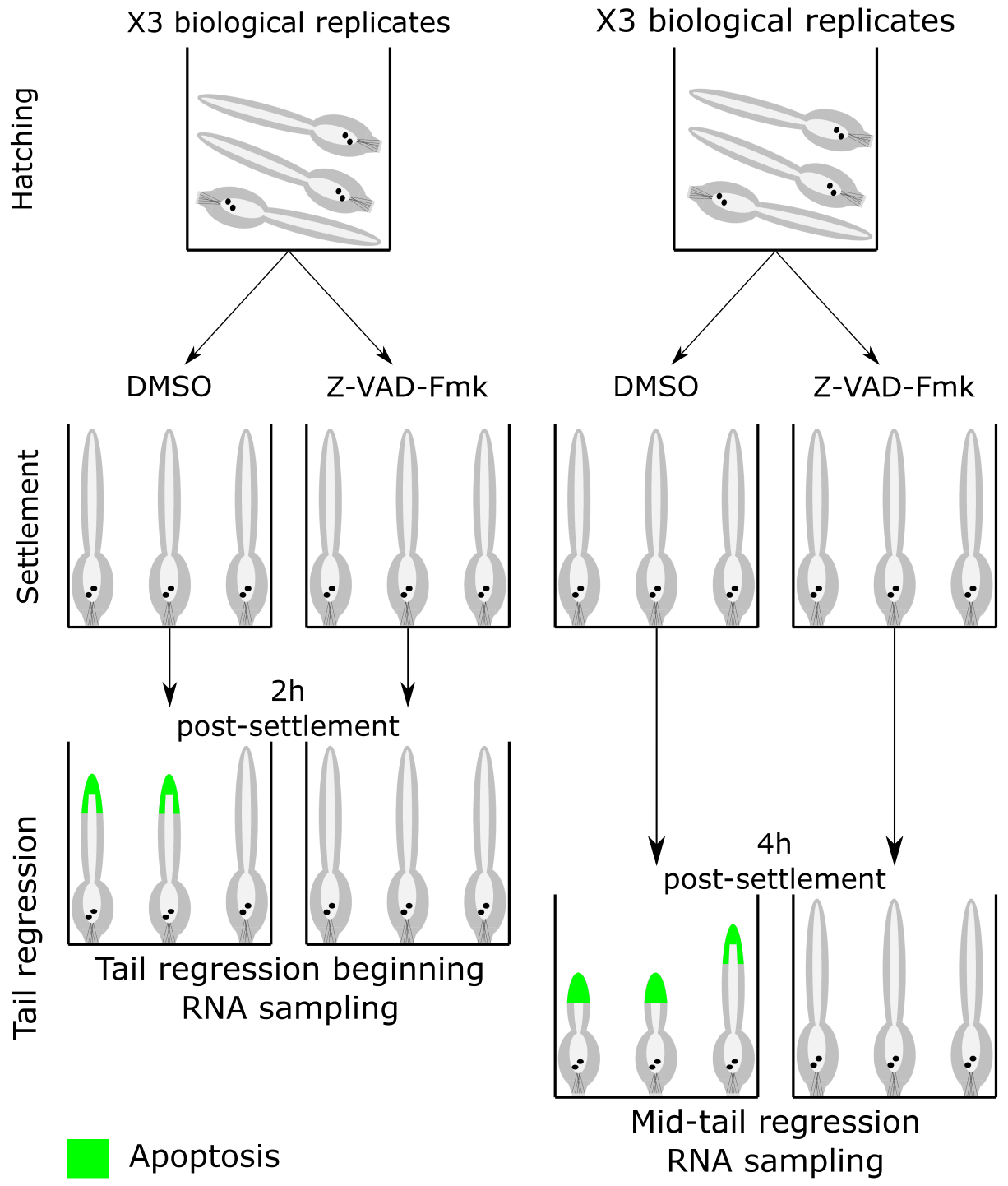

Supplement: Supplementary file 1 — Additional file 1: Supplementary Fig. 1. Experimental design. Six independent fertilisations were performed, and each of them was divided into two batches, in twelve petri dishes. After settlement, supernatant was discarded and replaced with filtered ASW containing DMSO (control) or Z-VAD-Fmk diluted in DSMO (treatment). RNA sampling was done at two time points according to the control: immediately after observation of tail regression beginning, and at mid-tail regression. [file 12860_2021_388_MOESM1_ESM.tif]
